# A placebo-controlled, double-blind, randomized, multicenter study to assess the effects of dronedarone 400 mg twice daily for 12 weeks on atrial fibrillation burden in subjects with permanent pacemakers

**DOI:** 10.1007/s10840-014-9966-z

**Published:** 2015-02-01

**Authors:** Michael D. Ezekowitz, Kenneth A. Ellenbogen, John P. DiMarco, Karoly Kaszala, Alexander Boddy, Gregory Geba P., Andrew Koren

**Affiliations:** 1 Sidney Kimmel Medical College, Thomas Jefferson University, 214 N. Ithan Ave, Villanova, PA USA; 2 Virginia Commonwealth University School of Medicine, Richmond, VA USA; 3 University of Virginia Health System, Charlottesville, VA USA; 4 Hunter Holmes McGuire VA Medical Center, Richmond, VA USA; 5 Sanofi-aventis US, Bridgewater, NJ USA

**Keywords:** Atrial fibrillation, Dronedarone, Pacemaker, Burden

## Abstract

**Purpose:**

Dronedarone is a benzofuran derivative with a pharmacological profile similar to amiodarone but has a more rapid onset of action and a much shorter half-life (13–19 h). Our goal was to evaluate the efficacy of dronedarone in atrial fibrillation (AF) patients using dual-chamber pacemakers capable of quantifying atrial fibrillation burden.

**Methods:**

Pacemakers were adjusted to optimize AF detection. Patients with AF burden >1 % were randomized to dronedarone 400 mg twice daily (BID) or placebo. Pacemakers were interrogated after 4 and 12 weeks of treatment. The primary endpoint was the change in AF burden from baseline over the 12-week treatment period. Patients with permanent AF, severe/recently decompensated heart failure, and current use of antiarrhythmic drugs were excluded. AF burden was assessed by a core laboratory blinded to treatment assignment.

**Results:**

From 285 patients screened, 112 were randomized (mean age 76 years, 60 % male, 84 % hypertensive, 65 % with sick sinus syndrome, 26 % with diabetes mellitus type II, 15 % with heart failure). Baseline mean (SEM) AF burden was 8.77 % (0.16) for placebo and 10.14 % (0.17) for dronedarone. Over the 12-week study period, AF burden compared to baseline decreased by 54.4 % (0.22) (*P* = 0.0009) with dronedarone and trended higher by 12.8 % (0.16) (*P* = 0.450) with placebo. The absolute change in burden was decreased by 5.5 % in the dronedarone group and increased by 1.1 % in the placebo group. Heart rate during AF was reduced to approximately 4 beats/min with dronedarone (*P* = 0.285). Adverse events were higher with dronedarone compared to placebo (65 vs 56 %).

**Conclusions:**

Dronedarone reduced pacemaker-assessed the relative AF burden compared to baseline and placebo by over 50 % during the 12-week observation period.

## Introduction

There is a need to develop safe and effective antiarrhythmic agents for the treatment of atrial fibrillation (AF) [1]. Dronedarone HCl, a benzofuran derivative with a pharmacologic profile similar to amiodarone [2–5], was approved by the Food and Drug Administration in 2009. In the USA, it is indicated to reduce the risk of hospitalization for AF in patients in sinus rhythm with a history of paroxysmal or persistent AF [6]. Results from key trials involving nearly 6000 patients demonstrated that dronedarone reduces AF recurrence and the composite of CV hospitalization and death in non-permanent AF [7, 8]. Dronedarone possesses all four Vaughan-Williams antiarrhythmic properties, with blockade of sodium channels, noncompetitive antiadrenergic activity, prolongation of the action potential and refractory periods, and calcium antagonist properties [2, 5, 6]. Compared to amiodarone, dronedarone lacks iodine, is less lipophilic, and has a shorter half-life (13–19 h vs ~58 days for amiodarone), thereby decreasing accumulation in tissue [5, 6, 9]. Dronedarone has a low systemic bioavailability, which increases up to 15 % when the drug is administered after a fatty meal. After repeated administration of 400 mg twice daily (BID), steady state is reached within 4 to 8 days [6, 10]. Dronedarone is metabolized by CYP3A enzyme system [6, 10].

The efficacy of antiarrhythmic drugs in patients with AF has been tested using the time to first recurrence of symptomatic AF [11–14]; AF events, however, frequently occur in clusters, thus questioning the validity of this endpoint [15–17]. In addition, time to first recurrence data fail to quantify AF burden (i.e., percent of time in AF), frequency, and number of episodes [12], all of which may carry a different prognosis. Asymptomatic episodes are common and complicate the assessment and may in fact represent the majority of AF episodes and may be associated with mortality and stroke risk [18, 19]. The availability of continuous monitoring permits documentation of frequency, duration, time of onset, and rate of AF as well as AF burden [17, 20, 21]. Patients with accepted indications for permanent pacemakers, particularly patients with sinus node disease, are at significant risk for AF, with prevalence rates of up to and over 50 % in some studies [22–24]. The Effects of Dronedarone on Atrial Fibrillation Burden in Subjects with Permanent Pacemakers (HESTIA; ClinicalTrials.gov number, NCT01135017) evaluates the impact of dronedarone on AF burden in patients with AF and permanent cardiac pacemakers.

## Methods

The intent of HESTIA was to enroll 290 AF patients with an interim analysis when 150 patients completed. Recruitment was slow and the study was terminated after enrollment of 112 patients. For entry, patients had to be at least 21 years of age with documented AF and sinus rhythm within the previous 6 months. Patients were included if one of the following CV risk factors were present: age ≥70 years, hypertension, diabetes mellitus, prior CV accident (stroke or transient ischemic attack) or systemic embolism, or M-mode or 2D echocardiography findings showing a left atrium diameter ≥50 mm or left ventricular ejection fraction less than ≤0.40 within the prior 12 months.

HESTIA was a placebo-controlled, double-blind, randomized, multicenter trial designed to assess the effects of dronedarone 400 mg BID versus placebo on AF burden in patients with a permanent pacemaker. Following patient consent, pacemakers were programmed to optimize atrial sensing. First, far-field R wave oversensing at maximum atrial sensitivity was assessed both during ventricular pacing and during ventricular sensing (if feasible). If far-field R wave oversensing was present, post-ventricular atrial blanking period (PVAB) was increased 25 ms beyond the longest far-field R wave signal. If there was no far-field R wave oversensing, PVAB was programmed to nominal setting. Final atrial sensitivity was set between 0.25 to 0.35 mV or above if needed (minimum allowable sensitivity was 0.75 mV). Patients were excluded if atrial sensing was <1 mV. The pacemakers were programmed to DDD or MVP mode and ventricular sensing was promoted by programming long AV delay if intrinsic conduction allowed. Mode switch rate was set to 200 bpm and the maximum number of bipolar atrial electrograms (EGMs) was stored for these episodes in the pacemakers. EGMs were reviewed as a quality control measure to ensure that they were due to atrial arrhythmias and that mode switches were accurate. Atrial overdrive pacing or atrial tachycardia therapies were programmed off. The study was conducted with support of a Pacemaker Core Laboratory (PERFUSE, Boston, MA) to ensure quality control. The core laboratory reviewed all interrogation reports and electrograms to ensure that pacemaker settings were consistent with protocol requirements and that pacemakers were accurately recording AF (i.e., atrial high rate episodes).

Patients were allowed treatment with concomitant medications based on dronedarone prescribing recommendations. Where possible, digoxin was either discontinued or halved. Treatment with Vaughan-Williams class I and III antiarrhythmics was prohibited. Use of amiodarone was prohibited within 4 weeks of screening.

Patients were randomized 1:1 to dronedarone or placebo and treated for 12 weeks. The trial was conducted in accordance with all applicable ethical and good clinical practice principles, including the Declaration of Helsinki. Written informed consent was obtained from all participants.

**Fig. 1:**
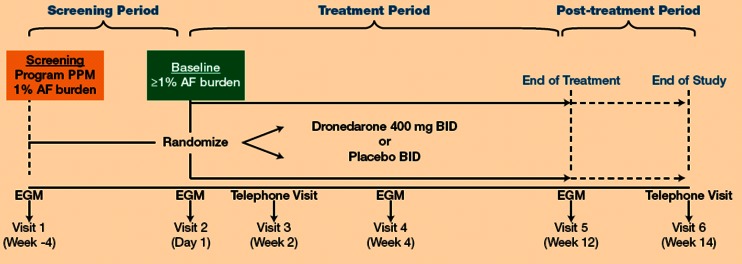
Trial design. *AF* atrial fibrillation, *BID* twice daily, *EGM* electrogram, *PPM* permanent pacemaker

All subjects had a dual-chamber pacemaker placed for an approved clinical indication from which AF burden was determined. There was an initial 4-week screening period (Fig. 1). AF burden was defined as arrhythmia time/24 h (e.g., 6 h of AF arrhythmia time would be 25 %). The AF burden had to be at least 1 % during the 4-week screening period off anti-arrhythmic therapy for study eligibility. For eligible patients, the AF burden during the screening period constituted the baseline AF burden. Pacemaker interrogations were also performed at 4 and 12 weeks after randomization. A final follow-up visit was performed 14 weeks after randomization.

The primary objective of this study was the effect of dronedarone on AF burden. Secondary objectives included the effects of dronedarone on ventricular rate during AF; patient-perceived AF burden; and symptom severity as reported by patients using the Atrial Fibrillation Severity Scale (AFSS). The number of electrical cardioversions and manual overdrive pacing events during treatment were collected.

An independent Data Monitoring Committee monitored safety, deaths, hospitalizations, and adverse events leading to drug discontinuation. Areas of special interest were congestive heart failure (CHF), diagnosis of interstitial lung disease, skin disorders, and peripheral neuropathy, the latter three are well known complications of amiodarone. CHF was assessed at screening and throughout the study. The planned sample size of 290 was estimated to have 70 % power to detect a reduction in mean AF burden of 30 % relative to the placebo group. The early termination of the study at 112 patients randomized reduced the power to detect treatment effects to under 50 %. The modified intent-to-treat (mITT) population included all patients who were randomized, treated with ≥1 dose of dronedarone, and had ≥1 post-baseline assessment of AF burden. The safety population was defined as the randomized population who received ≥1 dose of study medication. AF burden during the 12-week treatment period was analyzed in log-scale with adjustment for the baseline to compare AF burden between the dronedarone and placebo arms due to the non-normal distribution of AF burden. An analysis of covariance (ANCOVA) model was applied to the log-transformed AF burden data. The difference between treatment arms was estimated by the least squares mean method and was significant if the two-sided *p* value of the test was <0.05.

For secondary efficacy analyses, AF burden in the first 4 weeks and after 4 weeks of treatment was analyzed using the statistical method described for the primary efficacy variable and the average ventricular rate during AF episodes and the Atrial Fibrillation Severity Scale (AFSS) scores were analyzed without transformation using an ANCOVA model with treatment arm as a fixed effect term and the baseline value as a covariate. Electrical cardioversion (or overdrive pacing) was recorded.

## Results

**Fig. 2:**
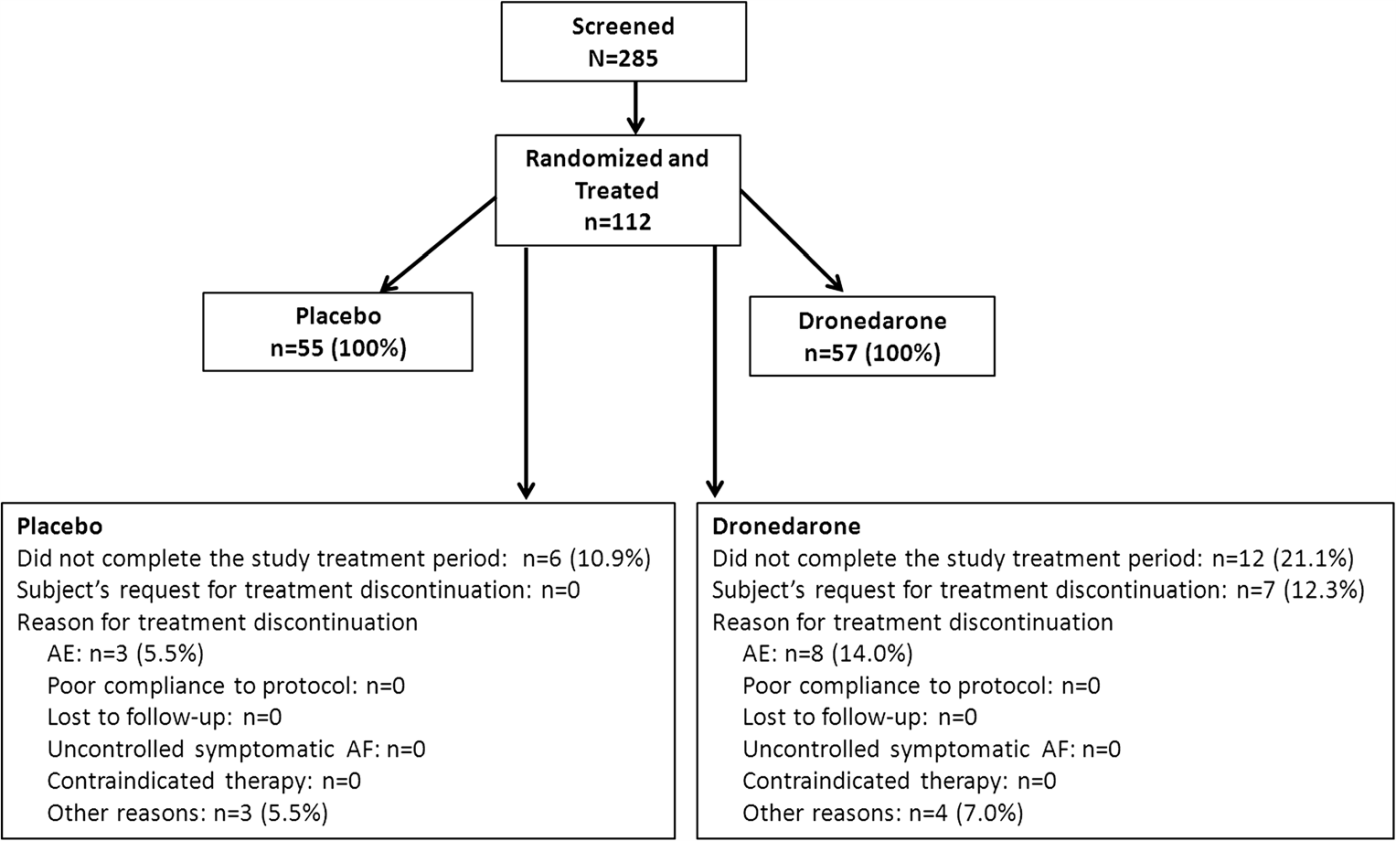
Patient disposition at end of study. *AFB* atrial fibrillation burden, *D/C* discontinuation, *ITT* intent to treat

Two hundred and eighty-five subjects were screened; 112 (Fig. 2 and Table 1) were randomized to receive treatment. Cardiovascular history of randomized subjects included hypertension in 84 %, sick sinus syndrome in 65 %, and congestive heart failure in 15 % of patients (Table 2). At randomization, 82 % were prescribed rate-lowering medications and 73 % oral anticoagulants (Table 3). Two subjects did not have a post-randomization interrogation of their pacemaker. Both were excluded from the primary efficacy analysis.

**Table 1:** Patient baseline demographics

	Placebo (*n* = 55)	Dronedarone (*n* = 57)	All (*N* = 112)
Age, mean (SD), years	74.5 (9.5)	77.3 (8.6)	75.9 (9.1)
Men, *n* (%)	38 (69.1)	29 (50.9)	67 (59.8)
Hispanic, *n* (%)	0	1 (1.8)	1 (0.9)
Race, *n* (%)			
Caucasian/White	50 (90.9)	54 (94.7)	104 (92.9)
Black	5 (9.1)	2 (3.5)	7 (6.3)
Asian/Oriental	0	1 (1.8)	1 (0.9)
Body mass index ≥30 kg/m^2^	24 (43.6)	22 (38.6)	46 (41.1)
Creatinine clearance, mean (SD), mL/min	73.2 (27.2)	66.1 (31.4)	69.6 (29.5)

*SD* standard deviation

**Table 2:** Cardiovascular history of randomized patients

	Placebo (*n* = 55)	Dronedarone (*n* = 57)	All (*N* = 112)
Hypertension	45 (81.8)	49 (86.0)	94 (83.9)
Sick sinus syndrome	37 (67.3)	36 (63.2)	73 (65.2)
Coronary artery disease	24 (43.6)	16 (28.1)	40 (35.7)
Syncope	15 (27.3)	20 (35.1)	35 (31.3)
Atrioventricular block above first degree	13 (23.6)	9 (15.8)	22 (19.6)
Congestive heart failure	6 (10.9)	11 (19.3)	17 (15.2)
Non-rheumatic valvular heart disease	7 (12.7)	9 (15.8)	16 (14.3)
Myocardial infarction	9 (16.4)	6 (10.5)	15 (13.4)
Ischemic stroke	6 (10.9)	5 (8.8)	11 (9.8)
Peripheral arterial disease	7 (12.7)	3 (5.3)	10 (8.9)
Transient ischemic attack	5 (9.1)	3 (5.3)	8 (7.1)
Supra-ventricular tachycardia other than atrial fibrillation/atrial flutter	3 (5.5)	4 (7.0)	7 (6.3)
Deep vein thrombosis	3 (5.5)	3 (5.3)	6 (5.4)
Stroke of unknown origin	1 (1.8)	4 (7.0)	5 (4.5)
Ischemic dilated cardiomyopathy	2 (3.6)	2 (3.5)	4 (3.6)
Torsades de pointe	1 (1.8)	0	1 (0.9)
Ventricular fibrillation	1 (1.8)	0	1 (0.9)

All data are expressed as *n* (%)

**Table 3:** Summary of baseline medications

	Placebo (*n* = 55)	Dronedarone (*n* = 57)	All (*N* = 112)
Beta blocking agents (except sotalol)	35 (63.6)	40 (70.2)	75 (67.0)
Angiotensin-converting enzyme inhibitors or angiotensin II receptor antagonists	33 (60.0)	24 (42.1)	57 (50.9)
Oral anticoagulant	38 (69.1)	44 (77.2)	82 (73.2)
Diuretics	25 (45.5)	21 (36.8)	46 (41.1)
Aspirin	26 (47.3)	19 (33.3)	45 (40.2)
Statins	34 (61.8)	32 (56.1)	66 (58.9)
Calcium antagonists with heart rate lowering effects	12 (21.8)	8 (14.0)	20 (17.9)
Digitalis	12 (21.8)	10 (17.5)	22 (19.6)
Other chronic antiplatelet therapy	10 (18.2)	5 (8.8)	15 (13.4)

All data are expressed as *n* (%)

Median duration of treatment was 12 weeks. Ninety-four subjects completed the study, and 18 subjects discontinued treatment due to the investigator’s or subject’s decision. Discontinuations due to an adverse event in the dronedarone group (14 %) exceeded those in the placebo group (6 %).

**Fig. 3:**
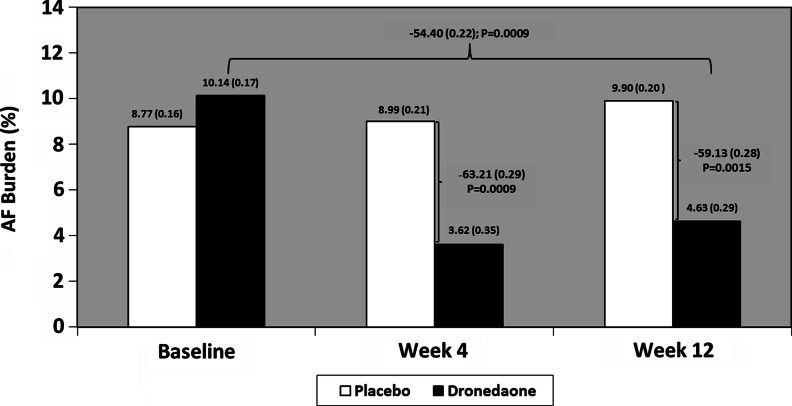
Baseline (week −4 to randomization), week 4, and week 12 (end of treatment) mean geometric AF burden. The primary efficacy result was the least mean square (LMS) difference in AF burden between placebo and dronedarone at week 12 (59.13 (0.28); *P* = 0.0015. Similar results were observed at week 4

**Fig. 4:**
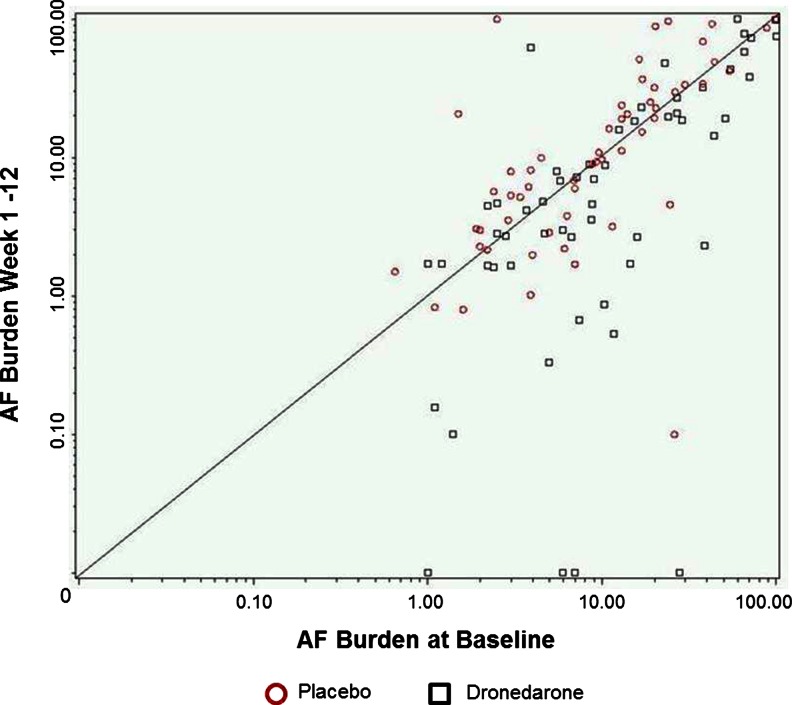
Plot of AF burden at baseline versus AF burden weeks 1–12. *Points above* the line represent increases in AF burden post-baseline and *points below* represent reductions in AF burden. Placebo is represented as *red circles* and dronedarone as *black squares*

Over the 12-week treatment period, mean AF burden increased from 8.8 % (0.16) to 9.9 % (0.20) (increase of 12.8 % (0.16); *P* = 0.450) in the placebo group and decreased from 10.1 % (0.17) to 4.6 % (0.29) (decrease of 54.4 % (0.22); *P* = 0.0009) in the dronedarone group. The absolute changes in AF burden from baseline increased by 1.1 % in the placebo group and decreased by 5.5 % in the dronedarone group. Compared to placebo, AF burden in the dronedarone group was decreased by 59.1 % (0.28 %) (*P* = 0.0015; Fig. 3). AF burden changes with dronedarone compared to placebo in secondary efficacy analyses were consistent with the overall result (weeks 1–4, 63.2 % (0.29) reduction, *P* = 0.0009; weeks 5–12, 60.3 % (0.30) reduction, *P* = 0.003). The distribution of baseline versus weeks 1–12 AF burden in treated patients is shown in Fig. 4.

The mean ventricular rate during AF was approximately 4 bpm lower in the dronedarone treatment group compared to placebo (*P* = 0.285). AFSS changes from baseline were not significantly different between groups (Table 4). Only one patient in the placebo group was cardioverted.

**Table 4:** Summary of Atrial Fibrillation Severity Scale (AFSS)

	Placebo (*N* = 55)	Dronedarone (*N* = 55)
AF burden		
Baseline mean (SD)	13.72 (4.59)	12.76 (4.62)
Post-baseline mean (SD)	13.30 (5.40)	12.48 (5.45)
Change from baseline, mean (SD)	−1.04 (3.22)	−0.68 (4.71)
Treatment difference, LS mean (SE)^a^	0.14 (0.981)	
*P* value	0.8902	
AF symptoms		
Baseline mean (SD)	8.47 (6.97)	8.36 (6.83)
Post-baseline mean (SD)	8.06 (7.11)	7.42 (7.48)
Change from baseline, mean (SD)	−0.28 (4.88)	−0.72 (5.66)
Treatment difference, LS mean (SE)^a^	−0.49 (0.980)	
*P* value	0.6216	

^a^From ANCOVA model

Treatment-emergent adverse events were common in both groups (dronedarone, 64.9 %; placebo, 56.4 %); most were gastrointestinal (Table 5). Treatment-emergent serious adverse events occurred in 14 patients (7 in each treatment group). Serious adverse events in the dronedarone group included congestive heart failure, peripheral neuropathy, ocular discomfort, presyncope, muscle rupture, and cardiomyopathy. More patients in the dronedarone group discontinued treatment due to adverse events compared with placebo (8 [14.0 %] vs 3 [5.5 %], respectively). There was no evidence of dronedarone-induced ventricular tachycardia and there were no deaths, strokes, or major bleeding events in the study.

**Table 5:** Treatment-emergent adverse events

	Placebo (*n* = 55)	Dronedarone (*n* = 57)
Overview of treatment-emergent adverse events		
Patients with any treatment-emergent adverse event	31 (56.4)	37 (64.9)
Patients with any treatment-emergent serious adverse event	7 (12.7)	7 (12.3)
Patients with any treatment-emergent adverse event leading to death	0	0
Patients with any treatment-emergent adverse event leading to permanent treatment discontinuation	3 (5.5)	8 (14.0)
Treatment-emergent adverse event of special interest		
Congestive heart failure	2 (3.6)	3 (5.3)
Treatment-emergent adverse events by primary system organ class		
Gastrointestinal disorders	10 (18.2)	17 (29.8)
Respiratory, thoracic, and mediastinal disorders	7 (12.7)	8 (14.0)
Nervous system disorders	6 (10.9)	7 (12.3)
Infections and infestations	8 (14.5)	6 (10.5)
Cardiac disorders	3 (5.5)	5 (8.8)
Metabolism and nutrition disorders	3 (5.5)	3 (5.3)
Vascular disorders	2 (3.6)	3 (5.3)
Psychiatric disorders	2 (3.6)	1 (1.8)
Eye disorders	1 (1.8)	1 (1.8)
Ear and labyrinth disorders	2 (3.6)	1 (1.8)
Skin and subcutaneous tissue disorders	2 (3.6)	1 (1.8)
Musculoskeletal and connective tissue disorders	4 (7.3)	1 (1.8)
Renal and urinary disorders	1 (1.8)	1 (1.8)
Immune system disorders	1 (1.8)	0
Hepatobiliary disorders	1 (1.8)	0

All data are expressed as *n* (%). Treatment-emergent adverse events include all patients randomized and that received at least one dose of study drug

## Discussion

The major finding was a 59 % relative reduction of AF burden with dronedarone compared to placebo (*P* = 0.0015). In the placebo group, the mean (geometric) AF burden increased from 8.8 to 9.9 % during the treatment period, whereas in the dronedarone group, the mean (geometric) AF burden was decreased from 10.1 to 4.6 %, an absolute reduction of 5.5 %. The effect was consistent over the 12-week duration of treatment. Average ventricular rate during AF was slightly (4 bpm) lower in the dronedarone group, but these differences were not statistically significant. There were no statistically significant differences in the AFSS. One patient was cardioverted.

Consistent with the known profile of dronedarone, there was an increase in gastrointestinal events. Serious adverse events and discontinuations due to adverse events were infrequent but occurred more often in the dronedarone group. There were no deaths in the study.

Two large multicenter, randomized, placebo-controlled studies evaluated the efficacy of dronedarone on AF recurrence [7, 8]. In the pooled analysis of the European Trial in Atrial Fibrillation or Flutter Patients Receiving Dronedarone for the Maintenance of Sinus Rhythm (EURIDIS, NCT00259428) and the American-Australian-African Trial with Dronedarone in Atrial Fibrillation or Flutter Patients for the Maintenance of Sinus Rhythm (ADONIS, NCT00259376), dronedarone significantly delayed the time to first recurrence of AF during the 12-month study period by approximately 25 % (*P* < 0.001 vs placebo), with an absolute difference in recurrence rate of ~11 % [8].

Use of pacemaker determined AF burden has been the subject of several prior studies and has potentially important clinical implications. A recent trial of patients with bradycardia and a history of AF receiving pacemakers indicated that total AF burden had a significant but weak correlation with cardiac hospitalization rates [25]. Although total AF burden is typically used as an endpoint in device trials such as the Atrial Therapy Efficacy and Safety Trial (ATTEST) [21, 26–29], it may also be a useful endpoint for antiarrhythmic medications. The Paroxysmal Atrial Fibrillation Study with Continuous Atrial Fibrillation Logging (PASCAL, NCT00389792) trial found up to a 74 % reduction in AF burden with budiodarone in an investigational dose ranging study [17].

The study had several limitations. First, study enrollment was below goals (290 planned vs 112 actual patients with interim analysis planned at 150 patients) because of the difficulty in finding non-permanent AF patients with at least 1 % AF burden on initial screening and during the 4-week baseline period. The study, however, was analyzed as originally planned with the primary endpoint achieving a highly significant (*P* = 0.0015) outcome. A second limitation is the use of mode switches to assess AF burden with the risks of under or oversensing. Efforts were made to mitigate this risk including use of a pacemaker core lab to ensure that pacemakers were appropriately set to study requirements and that pacemakers were accurately recording AF. Last, because HESTIA was placebo-controlled, patients with more severe AF symptoms were excluded. Low baseline AFSS burden and symptom scores reflect this and may have mitigated finding significant changes in the AFSS despite significant reductions in AF burden observed in the study.

The HESTIA study has important implications on design of future trials of antiarrhythmic drugs for AF. A post hoc calculation of study power based on our study findings was >95 %, underscoring the sensitivity of continuous ECG monitoring for assessing AF. In comparison, the EURIDIS and ADONIS studies required >500 patients each to detect a 25 % reduction in the rate of AF recurrence (at 12 months) with dronedarone compared to placebo with 90 % power. Continuous ECG monitoring also provides a more complete characterization of AF patterns compared with traditional time to first recurrence assessments, and also offers a sensitive method of detecting ventricular arrhythmias.

## Conclusion

The HESTIA study was designed to investigate the effects of dronedarone on AF burden in a population of patients experiencing intermittent episodes of AF. Prior to randomization, these patients were in AF an average of 10 % of the time. Dronedarone reduced the primary endpoint, time in AF, by slightly more than half (*P* < 0.005). This effect was consistent over the 12-week duration of treatment. Safety findings were consistent with the known profile of dronedarone.
